# Blubber biopsy biomarkers for baleen whales

**DOI:** 10.1093/conphys/coae080

**Published:** 2024-12-19

**Authors:** Ian A Bouyoucos

**Affiliations:** Department of Zoology, University of British Columbia, Vancouver, BC V6T 1Z4, Canada

**Figure 1:**
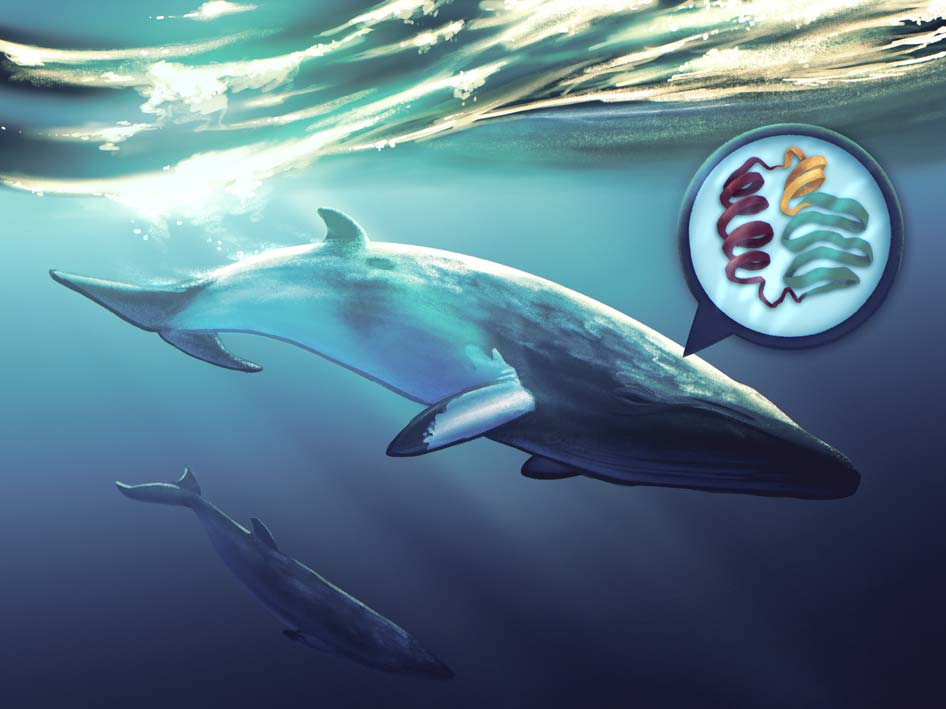
Illustration: Kaitlin Barham (kaitlin.barham@student.uq.edu.au)

Whales, dolphins and porpoises—collectively, cetaceans—are among the charismatic ocean giants that captivate and inspire future generations of marine biologists. In recent years, a draw to study cetaceans has, regrettably, been the need to urgently conserve them. As of 2023, the Cetacean Specialist Group of The International Union for Conservation of Nature identified that one in four cetacean species are threatened with extinction. By studying the health of wild cetaceans, scientists ultimately hope to better understand and effectively manage threats to populations (Fig. 1).

But how do researchers even go about studying how healthy a cetacean is in its natural habitat? From flying drones over humpback whales’ blowholes to collect samples from their breath to chasing down fresh scat from killer whales in dinghies, researchers are constantly innovating to overcome a simple, inconvenient truth: many cetaceans are enormous, and cannot be restrained and studied in a laboratory!

Joanna Kershaw and colleagues (Kershaw et al., 2024) are bringing cetacean conservation physiology into the 21st century with ‘omics’ techniques. Because many omics techniques are foreign to the world of cetacean conservation science, Kershaw and colleagues sought to add ‘shotgun proteomics’ to the conservation physiology toolbox. Proteomics describes the ‘proteome’, or all the proteins present in a sample, including potential biomarkers of the health of wild whales.

For their study, Kershaw and colleagues collected blubber biopsies from 10 female minke whales (*Balaenoptera acutorostrata*) in the Gulf of St. Lawrence (Canada) during their summer feeding season. The blubber biopsies were collected from a small, inflatable boat using a crossbow (no, not a shotgun) with a hollow-tipped arrow.

Blubber is a promising tissue to generate a snapshot of the health of wild cetaceans. Logistically, researchers can easily and safely collect it. Ethically, blubber biopsies only penetrate several centimetres, which is a fraction of how thick blubber is in many whales. Physiologically, blubber is a highly vascularized fatty tissue just under the skin, so blubber proteins reflect processes within the skin and fat, and proteins present in circulation from the blood.

To analyze their samples, the blubber biopsies underwent a standard shotgun proteomics pipeline. First, the blubber proteins are extracted and isolated from all the fatty tissue. Then the isolated proteins are digested into smaller peptides that are then separated and sequenced by liquid chromatography tandem mass spectrometry. Finally, the peptide sequences are aligned against known protein sequences for humans and assigned ‘biological process’ classifications based on their functions.

For the first time, Kershaw and colleagues demonstrated that shotgun proteomics can produce a valuable snapshot of the health of wild whales. Their approach allowed them to identify over 400 proteins. Most of the proteins identified were important for lipid metabolism, which means they are critical for managing energy stores in blubber. Indeed, Kershaw and colleagues posited that their approach could meaningfully reflect metabolic changes as minke whales fatten up in the Gulf of St. Lawrence each summer.

Kershaw and colleagues also identified many blubber proteins that are important for the immune system and antioxidant defence. Importantly, these blubber proteins are critical biomarkers for the overall health of wild minke whales. Ultimately, Kershaw and colleagues write that molecular techniques like theirs can contribute to marine mammal management and conservation; indeed, their study demonstrates that proteomic profiling of blubber biopsies is a promising approach to assessing the health of wild cetaceans.
